# Poor Ovarian Response to Gonadotrophins in PCOS Women after Laparoscopic Ovarian Drilling

**DOI:** 10.3390/medicina58020147

**Published:** 2022-01-19

**Authors:** Tanja Burnik Papler, Martin Stimpfel, Brina Kovacik, Eda Vrtacnik Bokal

**Affiliations:** 1 Department of Human Reproduction, Division of Obstetrics and Gynecology, University Medical Center Ljubljana, 1000 Ljubljana, Slovenia or martin.stimpfel@kclj.si (M.S.); eda.bokal@guest.arnes.si (E.V.B.); 2 Faculty of Medicine, University of Ljubljana, 1000 Ljubljana, Slovenia; 3 Obstetrics and Gynecology Hospital Kranj, 4000 Kranj, Slovenia; brina.zupancic@gmail.com

**Keywords:** PCOS, laparoscopic ovarian drilling, poor response, FSH level

## Abstract

*Background and Objectives:* Polycystic ovary syndrome (PCOS) is a major cause of anovulatory infertility, and ovulation induction is the first-line treatment. If this fails, laparoscopic ovarian drilling (LOD) is used to induce mono-ovulations. There have been implications, that LOD can cause destruction of ovarian tissue and therefore premature ovarian failure. Furthermore, unexpected poor ovarian response (POR) to gonadotrophins can occur in PCOS women after LOD. There have been reports about FSH receptor polymorphisms found in women with PCOS that are related to higher serum FSH levels and POR to gonadotrophins. *Materials and Methods:* In the present study, we retrospectively analyzed data of 144 infertile PCOS women that had LOD performed before IVF. *Results:* Thirty of included patients (20.8%) had POR (≤3 oocytes) to ovarian stimulation with gonadotrophins. Women with POR had significantly higher median levels of basal serum FSH (7.2 (interquartile range (IQR), 6.0–9.2) compared to women with normal ovarian response (6.0 (IQR, 5.0–7.4); *p* = 0.006). Furthermore, women with POR used a significantly higher median cumulative dose of gonadotrophins (1875 IU (IQR, 1312.5–2400) for ovarian stimulation compared to women with normal ovarian response (1600 IU (IQR, 1200–1800); *p* = 0.018). *Conclusion:* Infertile PCOS women who experience POR after LOD have significantly higher serum FSH levels compared to women with normal ovarian response after LOD. As these levels are still within the normal range, we speculate that LOD is not the cause of POR. We presume that women with PCOS and POR after LOD could have FSH-R genotypes associated with POR and higher serum FSH levels.

## 1. Introduction

Polycystic ovary syndrome (PCOS) is a frequent endocrinologic disorder that affects between 6–20% women of reproductive age [1]. Furthermore, around 80% of women with anovulatory infertility have PCOS [2]. In 2004, Rotterdam criteria for the establishment of the diagnosis of PCOS were determined [3]. According to these criteria, diagnosis of PCOS is confirmed when two out of three criteria are fulfilled: (1) oligo- and/or anovulation, (2) clinical and/or biochemical signs of hyperandrogenism, and (3) polycystic ovaries seen on ultrasound.

Ovulation induction is the first line of treatment for anovulatory infertility in women with PCOS. For this purpose, aromatase inhibitors—such as letrozole—or clomiphene citrate (CC) are used [4]. Clomiphene citrate is given for five consecutive days beginning on the third day of the menstrual cycle. If the ovulation fails after receiving a dosage of 150 mg per day for at least six consecutive cycles, the patient is defined as CC-resistant [5]. Laparoscopic ovarian drilling (LOD) or low-dose gonadotrophins for ovulation induction are considered as second-line treatments for CC-resistant patients [6]. LOD is a minimally invasive surgical procedure in which small perforations are made into the ovarian cortex by using monopolar/bipolar electrical current or laser [7]. In addition to LOD, transvaginal hydro laparoscopy (THL) has been developed as a less invasive alternative to conventional laparoscopy in infertile PCOS patients [8]. Sixty to 80% ovulation rates have been reported after LOD [9,10] and 64.1–82.9% after THL ovarian drilling [11].

In addition to being as effective as gonadotrophins for ovarian stimulation, LOD and THL offer an advantage over gonadotrophins, as they induce spontaneous mono-ovulations and therefore eliminate the risk of ovarian hyperstimulation syndrome and multiple pregnancies [5,12]. The mechanism by which mono-ovulations are induced is yet to be elucidated. LOD also improves the sensitivity of the ovaries towards subsequent FSH stimulation [13]. Despite its proven efficacy, LOD carries a hypothetical risk of premature ovarian failure [14], and patients suitable for LOD need to be carefully selected. A meta-analysis of Amer et al. [15] showed that AMH levels decline by 43% after LOD, whereas serum levels of FSH remain the same. Furthermore, Giampaolino et al. [16] reported a decline in serum AMH levels after THL ovarian drilling. For this reason, it has been suggested that this decline in AMH levels does not reflect a real decline in ovarian reserve but rather a normalization of pre-operative high levels. On the other hand, at our department, we have noticed poor ovarian response (POR) to gonadotrophin stimulation (≤3 oocytes) [17] during IVF procedures in some PCOS women after LOD. However, we do not know whether POR is truly a consequence of ovarian tissue destruction after LOD.

The aim of the present study was to compare PCOS women with normal ovarian response (NOR) and POR to gonadotrophin stimulation after LOD. Furthermore, we wanted to determine whether there are factors that could predict POR to ovarian stimulation (OS) in PCOS women who have previously had LOD. Uncovering such factors would enable us to decide which PCOS patients should undergo LOD and which should not. For this purpose, we retrospectively analyzed data of CC-resistant PCOS patients who underwent LOD and then OS during in-vitro fertilization (IVF) procedures.

## 2. Materials and Methods

We retrospectively analyzed data of 144 infertile PCOS women who had IVF procedures at our department between January 2011 and December 2016. All patients met the Rotterdam consensus criteria for the diagnosis of PCOS [3], and related disorders were excluded. Inclusion criteria were primary or secondary infertility; patent Fallopian tubes, confirmed by hysterosalpingography; normal semen analysis parameters of the patients’ partners according to the World Health Organization criteria; and age <35 years. All women had CC-resistant PCOS and underwent LOD before including them in IVF procedures.

Patients were divided into the following 2 groups: patients with NOR (4 to 15 retrieved oocytes) (*n* = 114) and patients with POR (3 or less oocytes retrieved) (*n* = 30) to OS.

We compared both groups of patients according to age, body mass index (BMI), serum FSH and LH level, type of gonadotrophin used for OS (urinary, recombinant), and daily and cumulative gonadotrophin dose used for OS by using the Mann–Whitney U test (Shapiro–Wilk test showed that data are not normally distributed). Because data are not normally distributed, they are presented as median with lower-quartile (Q1) and upper-quartile (Q3) values. Only data from the first IVF cycle were considered for analyses. We then tested for correlations between different variables by using Spearman’s correlation test. *p*-Values < 0.05 were considered statistically significant. Analyses were performed using the SPSS program.

## 3. Results

Of 144 women who had LOD performed in the past, 20.8% (*n* = 30) experienced POR to OS.

Table 1 shows the demographic, endocrine, and clinical characteristics of all women included in the study divided according to ovarian response to OS. There is a significant difference in serum FSH level, cumulative dose of gonadotrophins used for OS, number of retrieved oocytes, immature oocytes, and number of embryos between POR and NOR patients.

### 3.1. Statistical Analysis for All Patients Regardless of Ovarian Response

We compared clinical parameters and IVF outcomes between women where human menopausal gonadotrophin (hMG) or recombinant FSH (rFSH) was used for OS. A significantly lower daily and cumulative dose of FSH was used for OS in the rFSH group, whereas the number of oocytes retrieved was significantly higher in this group (Table 2).

Spearman’s correlation coefficient was calculated and there was a statistically significant positive correlation between BMI and cumulative gonadotrophin dose used for OS. A significantly negative correlation was found between BMI and FSH. Furthermore, there was a strong trend for a positive correlation between FSH and cumulative gonadotrophins dose and a significant positive correlation between the number of oocytes retrieved and the number of immature oocytes and embryos (Table 3).

### 3.2. Statistical Analysis—NOR Patients Only

There was a significantly positive correlation between the number of retrieved oocytes and the number of immature oocytes and embryos. Again, a significant negative correlation between BMI and FSH was seen as well as a positive correlation between FSH and cumulative gonadotrophin dose (Table 4).

### 3.3. Statistical Analysis—POR Patients Only

A significantly negative correlation between BMI and the number of embryos was seen. There was also a significantly positive correlation between the number of oocytes and the number of immature oocytes and embryos obtained (Table 5).

## 4. Discussion

In this retrospective study, a comparison of women with POR and NOR after OS, who had LOD performed in the past has, shown that POR women have significantly higher FSH levels. These levels, however, are still within the normal range for women of reproductive age.

Before the introduction of AMH and antral follicle count as markers of ovarian reserve, a widely used technique in reproductive medicine, as an indirect marker of ovarian reserve [5], were serum FSH levels measured between the third and fifth day of the menstrual cycle (early follicular phase).

It is known, however, that FSH only has a moderate sensitivity and specificity in predicting ovarian response to gonadotrophins, as its serum levels rise only when the ovarian reserve is severely compromised [18]. In the past, various cut-off values of serum FSH ranging from 10 to 15 IU/L have been recommended for predicting POR in IVF [19,20], but a generally accepted cut-off value above which one would expect POR has not yet been determined. As ovarian aging begins several years before FSH’s rise, normal FSH values do not exclude POR to OS [21]. Therefore, we can only predict POR to OS in women with extremely high levels of FSH.

The study of Su et al. [22] showed that the baseline LH/FSH ratio negatively correlates with live birth ratio in IVF procedures. They showed that LBR after fresh embryo transfer declines when baseline LH/FSH ratio is above 1. In our study, baseline LH/FSH ratio did not differ between POR and NOR group of patients; therefore, we could not use it for prediction of poor ovarian response to stimulation.

Serum levels of FSH were determined after LOD and prior to inclusion to IVF in the present study. Because LOD was performed in one institution and always according to the same surgical protocol, and all women had PCO morphology seen on transvaginal ultrasound, we may speculate that LOD is not directly responsible for POR to OS. We have shown, however, that serum FSH levels are not a reliable marker of ovarian reserve, as levels were within what is considered to be normal in our POR patients, but they still experienced POR to OS. In line with our findings are the results of the meta-analysis performed by Hendriks and co-workers [23], where they compared antral follicle count (AFC) and serum FSH levels for the prediction of ovarian response to OS in IVF procedures. They showed that FSH levels show intra- and inter-cycle variability and are influenced by several factors which limit its reliability. On the other hand, AFC is stable and directly shows ovarian reserve. They have concluded that AFC is a better predictor of ovarian response to OS than FSH.

One possible explanation for POR, in the present study, could be the presence of polymorphisms in the FSH-R gene. It has been shown that ovarian response to OS depends on the FSH-R genotype [24]. A study by Yan et al. [25] showed that certain FSH-R genotypes are related to an increased risk for POR. Furthermore, three different studies showed that women with and without PCOS having FSH-R Ser^680^ allele variant had higher basal FSH levels [26,27,28]. In women undergoing OS, this receptor variant is associated with lower E2 levels following OS, suggesting lower FSH-R sensitivity [29].

There were no significant differences in age and BMI between POR and NOR; therefore, these factors are not the reason for POR in the present study.

One of our presumptions in the present study was that POR patients received lower doses of gonadotrophins, and this was the reason for POR. However, this is not the case, as analyses have shown that the cumulative dose of gonadotrophins used for OS was significantly higher in POR patients. The daily dose of gonadotrophins was also higher in the POR group; however, it did not reach the threshold of statistical significance (*p* = 0.06). Our results are in line with the results of Tarlatzis et al. [30], who showed that the use of higher doses of gonadotrophins does not improve ovarian response.

Comparison of clinical parameters and IVF outcomes between rFSH and hMG has shown that daily and cumulative dose of rFSH used for OS is significantly higher if hMG is being used. Despite higher doses needed for OS, the number of retrieved oocytes was significantly lower in the hMG group. Our results are in line with previously published studies where the use of rFSH and hMG for OS was compared in women with PCOS [31], and there were no differences in the cumulative dose of gonadotrophins used, but a significantly higher number of oocytes were retrieved in the rFSH group.

We found a significant positive correlation between BMI and cumulative dose of gonadotrophins used for OS. Several previously published studies have shown a negative effect of increased body weight on ovarian response to OS [32,33,34,35,36]. In one of our previous studies, obese women required a 110% higher dose of rFSH for OS as compared to normal-weighing women [32]. A study by McCormick et al. [35] showed that women with PCOS and normal BMI required 30% less gonadotrophins for OS as compared to obese PCOS women. Besides that, 40% more oocytes were retrieved in women with normal BMI. In a study by Balen et al. [36], the amount of rFSH used in obese women was approximately 60% higher than in normal-weighing women. The reason for increased usage of gonadotrophins in women with increased body weight, however, has not yet been determined. It is possible that obesity affects the pharmacokinetics of gonadotrophins. Decreased sensitivity of women with increased body weight on exogenous gonadotrophins could be due to changes in their absorption, distribution, and metabolism because of increased body fat and surface [37,38,39].

A significantly negative correlation between BMI and FSH was found in the present study when data of all patients as well as only the NOR group were considered. The correlation was also negative for POR patients, but it did not reach statistical significance. A negative correlation between BMI and FSH has already been shown in normally cycling PCOS patients [40]. In this study, researchers hypothesized that body mass itself inhibits gonadotrophin secretion, and this could be true, as it is known that obese patients exhibit altered pulsatile gonadotropin secretion [41].

## 5. Conclusions

The present study shows that POR patients have significantly higher serum FSH levels compared to NOR patients; however, these values are within the normal range for women of reproductive age. Thus, we have again shown that FSH does not correctly predict response to OS when its levels are within the normal range. We have also shown that high BMI negatively impacts OS, as more gonadotrophins are needed for an ovarian response, which makes it more expensive. Furthermore, we have shown a negative correlation between BMI and serum levels of FSH. This could have an impact on ovarian response as well as the oocyte-maturation process. It would be therefore reasonable to advise overweight and obese PCOS women to reduce their body weight before inclusion in IVF procedures.

At this moment, we cannot tell what is the reason for POR in PCOS patients after LOD. As LOD was performed in only one institute and according to the same surgical technique, we cannot say that LOD itself is the reason for POR.

A personalized approach in PCOS treatment tailored according to the patient’s symptoms related to PCOS and her desire for pregnancy was already discussed in a review by Della Corte et al. [42]. Because the FSH-R genotype determines ovarian response to OS, and POR occurred in 20.8% of women undergoing OS in the present study, it would be reasonable to evaluate the FSH-R genotype in PCOS women before LOD. If we discovered genotypes related to POR, we would not perform LOD. Besides that, we would tailor OS according to the patient’s genetic background by adjusting gonadotrophin doses. Individualized OS could lead to a shorter duration of OS as well as lowering of the total amount of gonadotrophins used. Treatment would therefore be cheaper and more patient friendly.

## Figures and Tables

**Table 1:** Demographic, endocrine, and clinical characteristics of women with NOR and POR. Statistically significant differences are marked with an asterisk (*p*-value < 0.05).

	NOR (*n* = 114)	POR (*n* = 30)	*p*-Value
Age (years)	30 (27.75–33)	32 (29–34)	0.102
BMI (kg/m^2^)	25.3 (20.9–31.2)	23.5 (20.6–30.5)	0.798
FSH (IU/L)	6.0 (5.0–7.4)	7.2 (6.0–9.2)	0.006 *
LH (IU/L)	5.4 (3.4–8.2)	5.5 (2.9–9.9)	0.956
LH/FSH ratio	0.9 (0.6–1.3)	0.8 (0.5–1.2)	0.331
Daily gonadotrophin dose (all cycles)	150 (150–200)	175 (150–225)	0.109
Cumulative gonadotrophin dose (all cycles)	1600 (1200–1800)	1875 (1312.5–2400)	0.018 *
Daily gonadotrophin dose (only for cases with uFSH used)	225 (150–225)	225 (225–225)	0.442
Cumulative gonadotrophin dose (only for cases with uFSH used)	1875 (1631.25–2643.75)	2175 (1800–2868.75)	0.442
Daily gonadotrophin dose (only for cases with rFSH used)	150 (150–200)	150 (150–200)	0.810
Cumulative gonadotrophin dose (only for cases with rFSH used)	1175 (1200–1800)	1800 (1162.5–2250)	0.239
Nr of oocytes	8 (5–12)	1 (1–2)	<0.001 *
Nr of immature oocytes	1 (0–2)	0 (0–0)	<0.001 *
Nr of embryos	4 (2–6)	1 (0–1)	<0.001 *

NOR, normal ovarian response; POR, poor ovarian response; values reported as median with interquartile range (Q1–Q3).

**Table 2:** Comparison of hMG versus rFSH used for OS. Statistically significant differences are marked with an asterisk (*p*-value < 0.05).

	Urinary FSH (*n* = 16)	Recombinant FSH (*n* = 128)	*p*-Value
Age	33 (28.75–34.75)	30 (28–33)	0.076
BMI (kg/m^2^)	22.5 (20.8–29.9)	25.3 (20.8–31.2)	0.446
FSH (IU/L)	6.7 (5.2–10.2)	6.2 (5.2–7.8)	0.445
LH (IU/L)	3.0 (1.6–9.2)	5.5 (3.6–8.4)	0.050
LH/FSH ratio	0.5 (0.2–1.1)	0.9 (0.6–1.3)	0.008 *
Daily FSH dose (IU)	225 (168.75–225)	150 (150–200)	<0.001 *
Cumulative FSH dose (IU)	1987.5 (1800–2700)	1600 (1200–1950)	<0.001 *
Oocytes (*n*)	4 (1.25–9.25)	7 (4–11.75)	0.041 *
Immature oocytes (*n*)	0 (0–1)	0 (0–2)	0.673
Embryos (*n*)	2 (1–4.75)	4 (2–6)	0.073

Values reported as median with interquartile range (Q1–Q3).

**Table 3:** Spearman’s correlation for all patients. Statistically significant differences are marked with an asterisk (*p*-value < 0.05).

	BMI	FSH	Nr Oocytes	Nr Immature Oocytes	Nr Embryos	Cumulative Gonadotrophin Dose
**BMI**						
Correlation coefficient	1	−0.32	−0.01	0.08	−0.09	0.18
*p*-value		<0.001 *	0.853	0.413	0.278	0.029 *
**FSH**						
Correlation coefficient	−0.32	1	−0.17	−0.12	−0.10	0.16
*p*-value	<0.001 *		0.039 *	0.159	0.245	0.059
**Nr oocytes**						
Correlation coefficient	−0.01	−0.17	1	0.48	0.74	−0.10
*p*-value	0.853	0.039 *		<0.001 *	<0.001 *	0.163
**Nr immature oocytes**						
Correlation coefficient	0.08	−0.12	0.48	1	0.15	−0.11
*p*-value	0.413	0.159	<0.001 *		0.085	0.134
**Nr embryos**						
Correlation coefficient	−0.09	−0.10	0.74	0.15	1	−0.14
*p*-value	0.278	0.245	<0.001 *	0.085		0.075
**Cumulative gonadotrophin dose**						
Correlation coefficient	0.18	0.16	−0.10	−0.11	−0.14	1
*p*-value	0.029 *	0.059	0.163	0.134	0.075	

**Table 4:** Spearman’s correlation NOR patients. Statistically significant differences are marked with an asterisk (*p*-value < 0.05).

	BMI	FSH	Nr Oocytes	Nr Immature Oocytes	Nr Embryos	Cumulative Gonadotrophin Dose
**BMI**						
Correlation coefficient	1	−0.34	−0.03	0.11	−0.10	0.17
*p*-value		<0.001	0.760	0.238	0.276	0.076
**FSH**						
Correlation coefficient	−0.34	1	−0.03	−0.08	−0.05	0.19
*p*-value	<0.001 *		0.795	0.379	0.612	0.049 *
**Nr oocytes**						
Correlation coefficient	−0.03	−0.03	1	0.36	0.60	0.06
*p*-value	0.760	0.795		<0.001 *	<0.001 *	0.500
**Nr immature oocytes**						
Correlation coefficient	0.11	−0.08	0.36	1	−0.07	−0.02
*p*-value	0.238	0.379	<0.001 *		0.472	0.844
**Nr embryos**						
Correlation coefficient	−0.10	0.05	0.60	−0.07	1	−0.02
*p*-value	0.276	0.612	<0.001 *	0.472		0.838
**Cumulative gonadotrophin dose**						
Correlation coefficient	0.17	0.19	0.06	−0.02	−0.02	1
*p*-value	0.076	0.049 *	0.500	0.844	0.838	

**Table 5:** Spearman’s correlation POR patients. Statistically significant differences are marked with an asterisk (*p*-value < 0.05).

	BMI	FSH	Nr Oocytes	Nr Immature Oocytes	Nr Embryos	Cumulative Gonadotrophin Dose
**BMI**						
Correlation coefficient	1	−0.22	−0.27	−0.33	−0.39	0.30
*p*-value		0.244	0.156	0.071	0.034 *	0.111
**FSH**						
Correlation coefficient	−0.22	1	0.18	0.20	0.06	−0.13
*p*-value	0.244		0.335	0.292	0.766	0.493
**Nr oocytes**						
Correlation coefficient	−0.27	0.18	1	0.43	0.72	−0.09
*p*-value	0.156	0.335		0.018 *	<0.001 *	0.631
**Nr immature oocytes**						
Correlation coefficient	−0.33	0.20	0.43	1	0.20	−0.15
*p*-value	0.071	0.292	0.018 *		0.294	0.435
**Nr embryos**						
Correlation coefficient	−0.39	0.06	0.72	0.20	1	−0.06
*p*-value	0.034 *	0.766	<0.001 *	0.294		0.757
**Cumulative gonadotrophin dose**						
Correlation coefficient	0.30	−0.13	−0.09	−0.15	−0.06	1
*p*-value	0.111	0.493	0.631	0.435	0.757	
